# Standardising outcomes for clinical trials and systematic reviews

**DOI:** 10.1186/1745-6215-8-39

**Published:** 2007-11-26

**Authors:** Mike Clarke

**Affiliations:** 1 School of Nursing and Midwifery, Trinity College Dublin, 24 D'Olier Street, Dublin 4, Ireland

## Introduction

Fifteen years ago, what was to become OMERACT met for the first time in The Netherlands to discuss ways in which the multitude of outcomes in assessments of the effects of treatments for rheumatoid arthritis might be standardised. In *Trials*, Tugwell et al have described the need for, and success of, this initiative [1] and Cooney and colleagues have set out their plans for a corresponding initiative for ulcerative colitis [2]. Why do we need such initiatives? What's the problem? And are these and other initiatives the solution?

## What's the problem?

Every year, millions of journal articles are added to the tens of millions that already exist in the health literature, and tens of millions of web pages are added to the hundreds of millions currently available. Within these, there are many tens of thousands of research studies which might provide the evidence needed to make well-informed decisions about health care. The task of working through all this material is overwhelming enough, without then finding that the studies of relevance to the decision you wish to make all describe their findings in different ways, making it difficult if not impossible to draw out the relevant information. Of course, you might be able to find a systematic review, but even then there is no guarantee that the authors of that review will not have been faced with an insurmountable task of bringing together and making sense of a variety of studies that used a variety of outcomes and outcome measures.

These difficulties are great enough but the problem gets even worse when one considers the potential for bias. If researchers have measured a particular outcome in a variety of ways, (for example using different pain instruments filled in by different people at different times) they might not report all of their findings from all of these measures. Studies have highlighted this problem in clinical trials, showing that this selectivity in reporting is usually driven by a desire to present the most positive or statistically significant results [3]. This will mean that, where the original researcher had a choice, the reader of the clinical trial report might be presented with an overly optimistic estimate of the effect of an intervention and therefore be led towards the wrong decision.

In the 1990s, the potential scale of the problem of multiple outcome measures was highlighted in mental health by a comprehensive descriptive account of randomised trials in the treatment of people with schizophrenia. Thornley and

Adams identified a total of 2000 such trials, which had assessed more than 600 different interventions. However, these trials had included an even greater number of rating scales for mental health than the number of interventions: 640 [4]. The potential for biased reported and the challenges of comparing the findings of different trials of different interventions using different ways of measuring illness make the identification of effective, ineffective and unproven treatments for this condition especially difficult [5]. This is true whether the readers of the report of a clinical trial are trying to use it to inform their decisions, or whether they are trying to combine similar trials within a systematic review. Thornley and Adams, who had done the descriptive study of the large number of rating scales in mental health trials, were faced with this very problem in a review of chlorpromazine. They concluded that review with the following implications for research, "if rating scales are to be employed, a concerted effort should be made to agree on which measures are the most useful. Studies within this review reported on so many scales that, even if results had not been poorly reported, they would have been difficult to synthesise in a clinically meaningful way." [6].

## What's the solution?

If we want to choose the shortest of three routes between two towns, how would we cope if told that one is 10 kilometres and another is 8 miles? Doing that conversion between miles and kilometres might not be too much of a problem, but what if the third route was said to be 32 furlongs? Now, imagine that the measurements had all been taken in different ways. One came from walking the route with a measuring wheel, one from an estimate based on the time taken to ride a horse between the two towns and one from using a ruler on a map. To make a well informed choice we would want the distances to be available to us in the same units, measured in the same ways. Making decisions about health care should be no different. We want to compare and contrast research findings on the basis of the same outcomes, measured in the same ways.

Achieving this is not straightforward, but it is not impossible. Key steps are to decide on the core outcome measures and, in some cases, the core baseline variables, and for these to then be included in the conduct and reporting of research studies. One of the earliest examples is an initiative by the World Health Organisation in the late 1970s, relating to cancer trials. Meetings on the Standardization of Reporting Results of Cancer Treatment took place in Turin (1977) and in Brussels two years later. More than 30 representatives from cooperative groups doing randomised trials in cancer came together and their discussions led to a WHO Handbook of guidelines on the minimal requirements for data collection in cancer trials [7,8].

OMERACT has also grown by trying to reach a consensus among major stakeholders in the field of rheumatology [1] and the IMMPACT recommendations for chronic pain trials have arisen in a similar way [9]. Other approaches have included the use of literature surveys to identify the variety of outcome measures that have been used and reported, followed by group discussion. This is the case with low back pain [10], colon cancer [11] and an e-Delhi survey in maternity care [12].

Having developed these lists of outcomes measures, researchers need to use them and systematic reviewers need to build their reviews around them. These sets of standardised outcomes measures are not meant to stifle the development and use of other outcomes. Rather, they provide a core set of outcome measures, which researchers should use routinely. Researchers wishing to add other outcome measures in the context of their own trial would continue to do so but, when reporting their trial, selective reporting should be avoided through the presentation of the findings for both the core set and all additional outcome measures they collected. Furthermore, the use of the outcome measures in these core sets should not be restricted to research studies. They are also relevant within routine practice. If they are collected within such practice, they would help the provider and the receiver of health care to assess their progress and facilitate their understanding of the relevance to them of the findings of research.

Journals such as *Trials *can help by highlighting initiatives such as those discussed in rheumatology [1] and ulcerative colitis [2]. They should encourage researchers to report their findings for the outcome measures in the core sets, and provide them with the space to do so. This will allow readers and systematic reviewers to make best use of the reported trials.

## Conclusion

When there are differences among the results of similar clinical trials, the fundamental issues of interest to people making decisions about health care are likely to concern the interventions that were tested, the types of patient in the study, or both; not the different outcome measure used. The latter is important but if one remembers that the studies were probably not done to assess differences between the various ways of measuring outcomes, but, rather, differences between the interventions, the benefits of consistency become obvious. Achieving consistency is not something that can be left to serendipity. It will require consensus, guidelines and adherence. The papers in *Trials *and others mentioned in this commentary show how this might happen.

## Competing interests

I am the author of one of the papers on a core set of outcomes for healthcare research, which is cited in this paper.
